# Morphology and multi-gene phylogeny reveal a novel *Torula* (Pleosporales, Torulaceae) species from the plateau lakes in Yunnan, China

**DOI:** 10.3897/BDJ.11.e109477

**Published:** 2023-08-30

**Authors:** Sha Luan, Hong-Wei Shen, Dan-Feng Bao, Zong-Long Luo, Yun-Xia Li

**Affiliations:** 1 College of Agriculture and Biological Science, Dali University, Dali, China College of Agriculture and Biological Science, Dali University Dali China; 2 Center of Excellence in Fungal Research, Mae Fah Luang University, Chiang Rai, Thailand Center of Excellence in Fungal Research, Mae Fah Luang University Chiang Rai Thailand; 3 School of Science, Mae Fah Luang University, Chiang Rai, Thailand School of Science, Mae Fah Luang University Chiang Rai Thailand

**Keywords:** 1 new species, lignicolous freshwater fungi, phylogeny, taxonomy

## Abstract

**Background:**

During an investigation into lignicolous freshwater fungi from the plateau lakes in Yunnan Province, China, two fresh collections of *Torula* taxa were collected and examined morpholgically.

**New information:**

*Torulaluguhuensis* is characterised by: conidiophores which are semi-macronematous mononematous, erect, septate, smooth, slightly flexuous and pale brown; conidiogenous cells which are holoblastic, mono- to polyblastic, integrated, terminal, terminal or intercalary in conidial chains, doliiform and pale brown; conidia which are branched chains, acrogenous, straight or slightly curved, dark brown to blackish, pale brown or subhyaline at apex, 1–3 septate, strongly constricted at the septa, verruculose or finely echinulate and rounded at both ends. A new species was introduced, based on morphological and phylogenetic analysis of combined ITS, LSU, RPB2 and TEF sequence data. Detailed descriptions and illustrations are provided, with an updated phylogenetic tree depicting intergeneric relationships within the Torulaceae.

## Introduction

Torulaceae was introduced by Corda (Sturm 1829) with *Torula* as the type. The family is known only by the asexual morph which is characterised by: mostly immersed mycelium, erect, micro- or macronematous, straight or flexuous, subcylindrical conidiophores with or without apical branches and doliiform to ellipsoid or clavate, brown, smooth to verruculose and mono- to polyblastic conidiogenous cells and subcylindrical, phragmosporous, acrogenous, brown, dry and smooth to verrucose conidia that are characteristically produced in branched chains (Crous et al. 2015, Su et al. 2016, Hyde et al. 2016, Li et al. 2017, Li et al. 2020). Currently, six genera, viz. *Cylindrotorula*, *Dendryphion*, *Neopodoconis*, *Neotorula*, *Rutola* and *Torula* are accommodated in Torulaceae (Crous et al. 2015, Su et al. 2016, Li et al. 2016, Su et al. 2018, Crous et al. 2020, Qiu et al. 2022).

*Torula* was introduced by Persoon (1795) and is typified by *T.herbarum*. Members in this genus are hyphomycetes and characterised by superficial dark colonies, terminal or lateral, monoblastic or polyblastic conidiogenous cells with a basally thickened and heavily melanised wall, a thin-walled apex and medium to dark brown conidia in branched chains (Crane and Miller 2016). *Torula* has been investigated as an interesting source of secondary metabolites. For example, a new dechlorinated aromatic lactone produced by *Torula* sp. (YIM DT 10072) exhibited antibacterial activity against *Staphylococcusaureus* (Chunyu et al. 2018). Herbarin, dehydroherbarin and o-methylherbarin have been extracted from *Torulaherbarum* (Narasimhachari and Gopalkrishnan 1974).

Yunnan is an inland province at a low latitude and high elevation, lying between 21°09’–29°15’ N and 97°32’–106°12’ E in south-western China, an area which is rich in freshwater resources. The nine major plateau lakes represented by Dianchi Lake, Erhai Lake and Fuxian Lake are major features of Yunnan. Abundant freshwater lake resources provide a favourable environment for the occurrence of lignicolous freshwater fungi (Shen et al. 2022). The studies of lignicolous freshwater fungi in Yunnan are mainly focused on lotic habitats (Su et al. 2016, Luo et al. 2019). At present, only a limited number of early studies have explored the diversity of lignicolous freshwater fungi in Dianchi Lake and Fuxian Lake (Cai et al. 2002, Luo et al. 2004). Presently, we are conducting systematic research on lignicolous freshwater fungi from plateau lakes in Yunnan Province. In this study, two *Torula* species were collected from Luguhu Lake and their phylogenetic relationships were analysed, based on molecular sequence data.

## Materials and methods

### Isolation and morphological study of strain

Submerged decaying woods were collected from Luguhu Lake, Yunnan Province and brought to the laboratory in zip-lock plastic bags. The samples were incubated in plastic boxes lined with moistened tissue paper at room temperature for one week and examined by methods following Luo et al. (2018). Micromorphological characters were observed using an Optec SZ 760 compound stereomicroscope. Temporarily prepared microscope slides were placed under a Nikon ECLIPSE Ni-U compound stereomicroscope for observation and micro-morphological-photography. The morphology of colonies on native substrates were photographed with a Nikon SMZ 1000 stereo zoom microscope.

Single spore isolations were carried out following the methods described by Senanayake (2020). Germinating conidia were transferred aseptically to PDA plates supplemented with 0.5 mg/l of Amoxicillin and grown at room temperature.

Specimens were deposited in the Herbarium of the Kunming Institute of Botany, Chinese Academy of Sciences (KUN-HKAS), Kunming, China. The cultures were deposited in China General Microbiological Culture Collection Center (CGMCC) and Kunming Institute of Botany Culture Collection (KUNCC). The MycoBank number was registered at https://www.mycobank.org.

### DNA extraction, PCR and sequencing

Fungal mycelium was scraped from the surface of colonies grown on PDA at room temperature. The Trelief^TM^ Plant Genomic DNA Kit (TSP101-50) was used to extract DNA from the ground mycelium according to the manufacturer’s instructions. The primers used for PCR amplification were ITS = ITS5/ITS4 (White et al. 1990), LSU = LR0R/LR5 (Vilgalys and Hester 1990), TEF-α = 983F/2218R and RPB2 = fRPB2-5F/fRPB2-7cR (Liu et al. 1999). The final volume of the PCR reaction was 25 μl and contained 12.5 μl of 2× Power Taq PCR MasterMix, (20 mM Tris-HCL pH 8.3, 100 mM KCl, 3 mM MgCl_2_, stabiliser and enhancer), 1 μl of each primer (10 μM), 1 μl genomic DNA extract and 9.5 μl deionised water. The PCR of ITS genes was processed as follows: 94℃ for 3 minutes, followed by 35 cycles of denaturation at 94℃ for 30 seconds, annealing at 56℃ for 50 seconds, elongation at 72℃ for 60 seconds and final extension at 72℃ for 10 minutes. The LSU and TEF genes were processed as follows: 94℃ for 3 minutes, followed by 35 cycles of denaturation at 94℃ for 30 seconds, annealing at 55℃ for 50 seconds, elongation at 72℃ for 60 seconds and final extension 72℃ for 10 minutes. The RPB2 gene region was amplified with an initial denaturation of 95℃ for 5 minutes, followed by 40 cycles of denaturation at 95℃ for 60 seconds, annealing at 52℃ for 2 minutes, elongation at 72℃ for 90 seconds and final extension at 72℃ for 10 minutes.

PCR amplification was confirmed on 1% agarose electrophoresis gels stained with ethidium bromide. Purification and sequencing of PCR products were sent for sequencing at Tsingke Biological Engineering Technology and Services Company, Yunnan, China. The sequences were deposited in the GenBank database at the National Center for Biotechnology Information (NCBI) and the accession numbers are listed in Table 1.

### Sequencing and sequence alignment

Sequences were assembled with BioEdit (Hall 1999) and those with high similarity indices were determined from a BLAST search to find the closest matches with taxa in *Torula* and from recently-published data (Li et al. 2020, Li et al. 2023, Tian et al. 2023). Aligned sequences of each loci (ITS, LSU, RPB2 and TEF) were combined and manually improved using BioEdit v.7.0.5.2 (Hall 1999). All consensus sequences and the reference sequences were automatically aligned with MAFFT (Katoh and Standley 2013). Additionally, sequence trimming was performed with trimAl v.1.2 with default parameters (http://trimal.cgenomics.org for specific operation steps) (Capella-Gutiérrez et al. 2009) and combined using SequenceMatrix (Vaidya et al. 2011). Ambiguous regions were excluded from the analysis and gaps were treated as missing data. FASTA alignment formats were changed to PHYLIP and NEXUS formats using the website Alignment Transformation Environment (ALTER) (http://sing.ei.uvigo.es/ALTER/).

### Phylogenetic analyses

Maximum Likelihood (ML) analysis was performed by setting RAxML-HPC2 on XSEDE (8.2.12) (Stamatakis 2006, Stamatakis et al. 2008) in the CIPRES Science Gateway (Miller et al. 2010) (http://www.phylo.org/portal2) using the GTR+GAMMA model with 1000 bootstrap repetitions. Bayesian analyses were performed in MrBayes 3.2.6 (Ronquist et al. 2012) and the best-fitting model of sequences evolution was estimated via Capella-Gutiérrez 2.2 (Guindon and Gascuel 2003, Darriba et al. 2012, Ronquist et al. 2012). The Markov Chain Monte Carlo (MCMC) sampling approach was used to calculate posterior probabilities (PP) (Rannala and Yang 1996). Bayesian analyses of six simultaneous Markov chains were run for 5 M generations and trees were sampled every thousand generations. Phylogenetic trees were visualised using FigTree v.1.4.0 (http://tree.bio.ed.ac.uk/software/figtree/), while editing and typesetting were achieved using Adobe Illustrator (AI) (Adobe Systems Inc., United States).

## Taxon treatments

### Torula luguhuensis

S. Luan, H.W. Shen & Z.L. Luo sp. nov.

9F1D77ED-7BB0-5D43-AB8A-9C67661B876B

MB 848773

#### Materials

- **Type status:** Holotype. **Occurrence:** recordedBy: Sha Luan; occurrenceID: B75E1EC7-0546-585A-A29A-1577106D0D0B; **Taxon:** scientificName: *Torulaluguhuensis*; kingdom: fungi; phylum: Ascomycota; class: Dothideomycetes; order: Pleosporales; family: Torulaceae; genus: Torula; **Location:** waterBody: Luguhu Lake; locationRemarks: China, Yunnan Province, submerged decaying wood in Luguhu Lake; verbatimLatitude: 27°44′13.59″N; verbatimLongitude: 100°49′04.72″E; **Identification:** identifiedBy: Sha Luan; **Event:** habitat: freshwater, submerged decaying wood; **Record Level:** collectionID: LGH H 6-43-1; collectionCode: L335

#### Description

**Figure 1.**
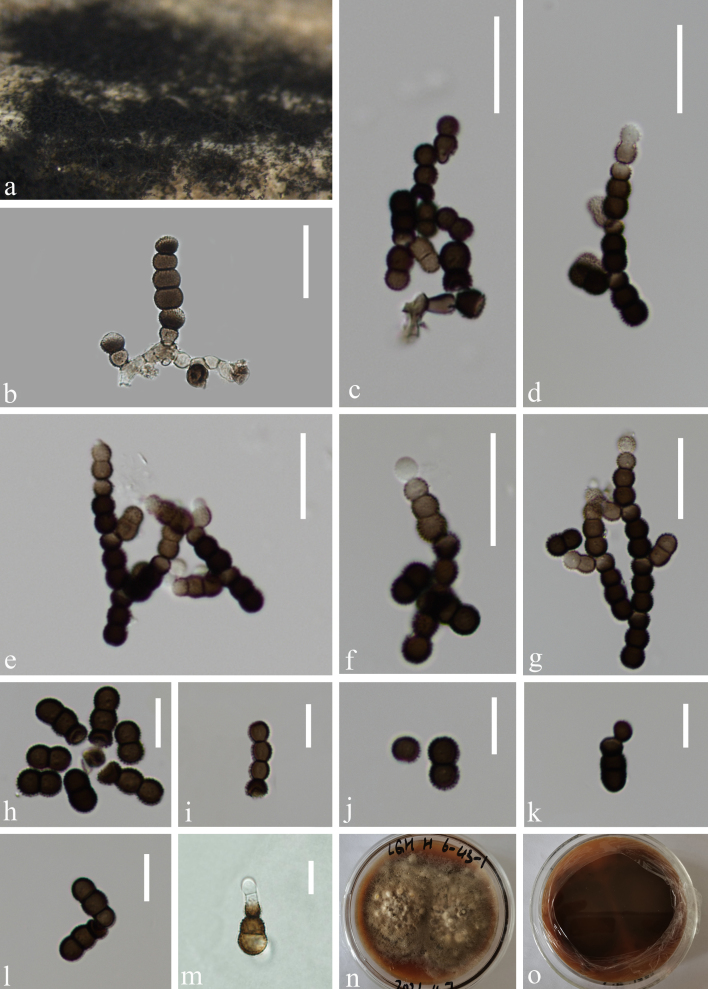
*Torulaluguhuensis* (KUN-HKAS 124588, holotype). **a** Colonies on decaying wood; **b, c** Conidiophores with conidia; **d-l** Conidia; **m** Germinating conidium; **n, o** Colonies on PDA from surface and reverse. Scale bars: b-g 20 μm, h-m 10 μm.

Saprobic on submerged decaying wood (Fig. 1a). **Sexual morph**: Undetermined. **Asexual morph**: Colonies effuse on nature substrate, scattered, velutinous, dark brown to black. Mycelium immersed to superficial, composed of hyaline, becoming brown closer to fertile region, septate, branched hyphae. Conidiophores semi-macronematous mononematous, erect, septate, smooth, slightly flexuous, pale brown (Fig. 1b and c). Conidiogenous cells holoblastic, mono- to polyblastic, integrated, terminal, terminal or intercalary in conidial chains, doliiform, pale brown. Conidia in branched chains, dry, acrogenous, straight or slightly curved, more or less cylindrical, dark brown to blackish, pale brown or subhyaline at apex, 1–3 septate, strongly constricted at the septa, verruculose or finely echinulate, rounded at both ends, easily separating, 12–18 μm (\begin{varwidth}{50in}\begin{equation*}
            x ̅
        \end{equation*}\end{varwidth} = 15 μm, SD = 3, n = 60) long, 6–8 μm (\begin{varwidth}{50in}\begin{equation*}
            x ̅
        \end{equation*}\end{varwidth} = 7 μm, SD = 1, n = 60) wide (Fig. 1d-m).

##### Culture characteristics

Conidia germinating on PDA within 12 hours and germ tubes produced from the apex. Colonies growing on PDA, reaching 10 cm in 15 days at 24℃, mycelium partly superficial, partly immersed, hairy, with regular edge, maroon to yellowish-brown (Fig. 1o and p) .

##### Material examined

China, Yunnan Province, submerged decaying wood in Luguhu Lake, 100°49′04.72″E, 27°44′13.59″N, March 2021, Sha Luan, *Torulaluguhuensis* (KUN-HKAS 124588, holotype), ex-type culture, CGMCC 3.24256 = KUNCC 22–12427.

#### Etymology

Referring to Luguhu Lake, China, where the fungus was collected.

#### Notes

In the multigene phylogenetic analysis, *Torulaluguhuensis* clustered with *T.aquatica* (MFLUCC 16–1115, DLUCC 0550) with 100% ML and 1.00 PP support. *Torulaluguhuensis* resembles *T.aquatica* in having macronematous or semi-macronematous, erect conidiophores and verruculose conidia (Su et al. 2018). However, *Torulaluguhuensis* differs from *T.aquatica* in having larger conidia (12–18 × 6–8 vs. 9–14 × 5–6 μm). A comparison of RPB2, ITS and LSU nucleotides between *T.luguhuensis* and *T.aquatica* showed 48/775 bp (6.2%), 5/433 bp (1.2%) and 3/796 bp (0.3%) differences with no gaps, respectively. Based on morphological and phylogenetic analysis, we introduce *T.luguhuensis* as a new species.

### Torula submersa

W.H. Tian, Y.P. Chen & Maharachch J. Fungi 2023

3CA4EDE0-4B82-55A0-99FA-97DBBA75CD07

MB 847013

#### Description

**Figure 2.**
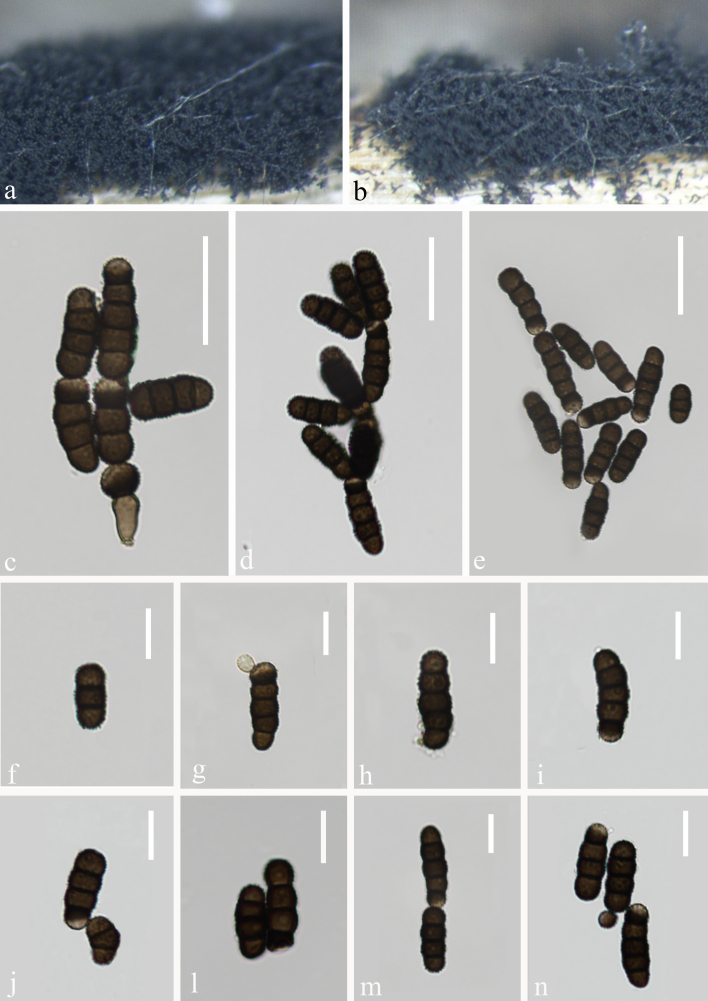
*Torulasubmersa* (HKAS 126510). **a-b** Colonies on decaying wood; **c** Conidiophores with conidia; **d-n** Conidia. Scale bars: c-e 20 μm, f-n 10 μm.

Saprobic on submerged decaying wood (Fig. 2a, b). **Sexual morph**: Undetermined. **Asexual morph**: Colonies effuse on nature host, black, friable. Mycelium immersed to superficial, composed of septate, pale brown, branched hyphae. Conidiophores macronematous, mononematous, erect, smooth, straight or slightly flexuous, dark brown to pale brown (Fig. 2c). Conidiogenous cells polyblastic, terminal, dark brown, pale brown at apex, on conidiophores, minutely verruculose, doliiform to subglobose. Conidia solitary or in branched chains, acrogenous, simple, phragmosporous, dark brown, pale brown at apex, 2–4-septate, constricted at the septa, verruculose or finely echinulate, rounded at both ends, easily separating; 14–20 μm (\begin{varwidth}{50in}\begin{equation*}
            x ̅
        \end{equation*}\end{varwidth} = 17 μm, SD = 3, n = 60) long, 6–8 μm (\begin{varwidth}{50in}\begin{equation*}
            x ̅
        \end{equation*}\end{varwidth} = 7 μm, SD = 1, n = 60) wide (Fig. 2d-n).

##### Culture characteristics

Conidia germinating on PDA within 10 hours and germ tubes produced from the apex. Colonies growing on PDA, reaching 10 cm in 15 days at 24℃. Colonies were raised in the middle, velvety on the surface and had a white centre fading to yellowish-brown, reverse, yellowish-brown in centre and white edges.

##### Material examined

China, Yunnan Province, submerged decaying wood in Luguhu Lake, 100°49′08.33″E, 27°39′39.24″N, March 2021, Yan Tao, L147 (KUNCC 22–12426).

#### Notes

**Figure 3.**
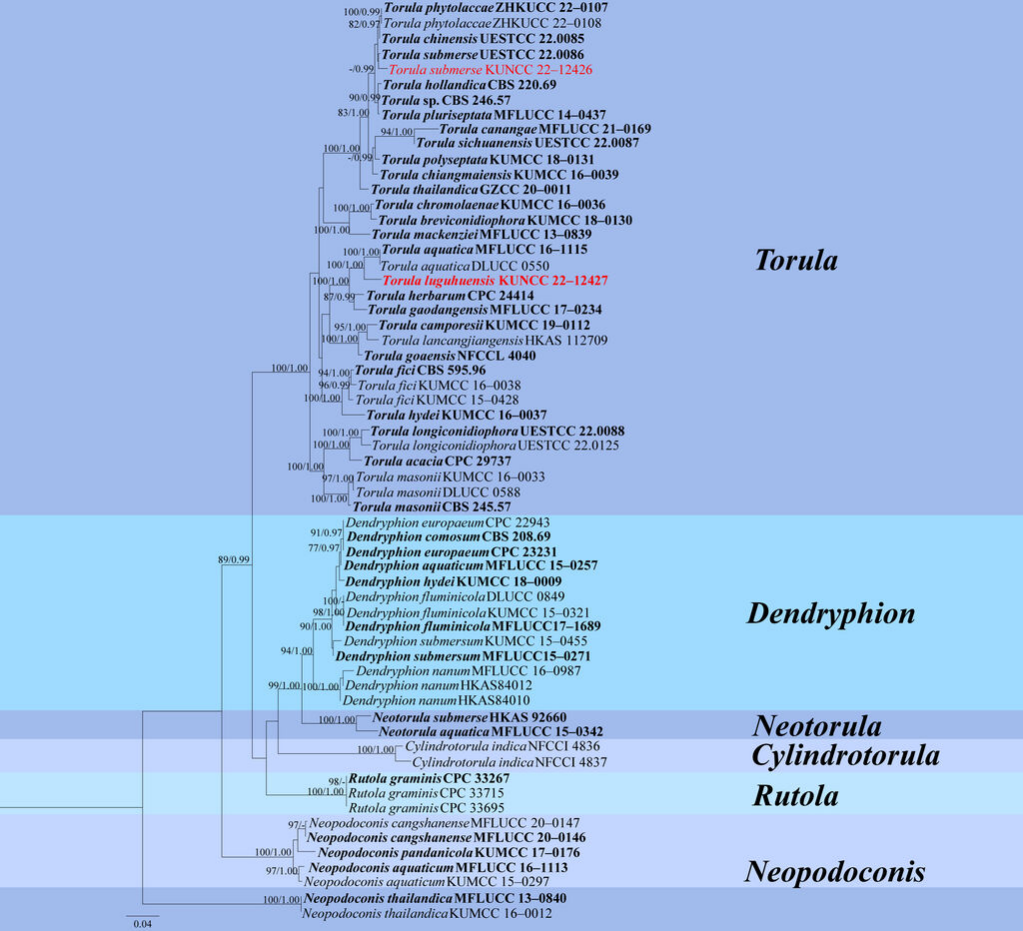
Phylogram generated from Maximum Likelihood analysis, based on combined ITS, LSU, RPB2 and TEF sequence data for species of Torulaceae. RAxML bootstrap support values equal to or greater than 75% are given before the forward slash. Branches with Bayesian posterior probabilities equal to or higher than 0.95 are given after the forward slash.

*Torulasubmersa* was introduced by Tian et al. (2023), collected on a submerged decaying branch from Sichuan Province. In this study, a fresh collection was made on submerged decaying wood in Luguhu Lake, Yunnan Province. Phylogenetic analysis showed that our collection (KUNCC 22–12426) sistered with *T.submersa* (*Fig. 3*). Morphological characteristics of our new collection are consistent with *Torulasubmersa* (Tian et al. 2023). Based on morphological characteristics and phylogenetic analysis, we, therefore, identified our new collection as *Torulasubmersa*, which was collected from a lentic freshwater habitat for the first time.

## Discussion

Species of *Torula* are quite similar in morphology and most species lack DNA sequence data to support their phylogenetic relationships (Crous et al. 2015). This causes taxonomic confusion and some species may have been misidentified. Some *Torula* species may be the same or belong to other genera and their taxonomic statuses have to be further investigated (Crane and Miller 2016). In addition to the morphological examination, DNA-based phylogenetic analysis should be performed for more *Torula* species. Herein, we combined multi-loci phylogenetic analysis and morphological characterisation to introduce one new species which contributes to the taxonomy for the genus and addition of DNA sequence in databases.

Taxonomic research on Torulaceae in China is mainly concentrated in the south-western region and commonly found in freshwater habitats (Hyde et al. 2016, Su et al. 2016, Su et al. 2018, Qiu et al. 2022, Tian et al. 2023). There are four genera of Torulaceae viz. *Dendryphion*, *Neopodoconis*, *Neotorula* and *Torula* which are reported from freshwater habitats in China. In this study, a checklist of Torulaceae species in China is provided. Torula is commonly found on submerged decaying wood in freshwater environments with most species having been isolated from lotic water (Table 2). In our study, we found a new species in a lake in Yunnan Province. Presumably there could be other new species in these habitats and it is necessary to investigate lignicolous freshwater fungi in other lakes in Yunnan. A checklist of *Torulaceae* species from freshwater habitats in China is shown in Table 2 below.

## Supplementary Material

XML Treatment for Torula luguhuensis

XML Treatment for Torula submersa

## Figures and Tables

**Table 1.** Taxa used in the phylogenetic analysis and their corresponding GenBank accession numbers. The newly-generated sequences and the ex-type strains are in bold.

**Species**	**Culture/Voucher**		**GenBank accession numbers**			
			**ITS**	**LSU**	**RPB2**	**TEF**
***Torulaacaciae***		CPC 29737	NR 155944	NG 059764	KY173594	-
*Torulaaquatica*		DLUCC 0550	MG208166	MG208145	MG207976	MG207996
***Torulaaquatica***		MFLUCC16–1115	MG208167	MG208146	MG207977	-
***Torulaluguhuensis***		**KUNCC 22**–**12427**	**OQ729758**	**OQ947766**	**OQ999002**	**OQ999004**
***Torulabreviconidiophora***		KUMCC 18–0130	MK071670	MK071672	-	MK077673
*Torulacamporesii*		KUMCC 19–0112	MN507400	MN507402	MN507404	MN507403
***Torulachiangmaiensis***		KUMCC 16–0039	MN061342	KY197856	-	KY197876
***Torulachromolaenae***		KUMCC 16–0036	MN061345	KY197860	KY197873	KY197880
***Torulafici***		CBS 595.96	KF443408	KF443385	KF443395	KF443402
*Torulafici*		KUMCC 15–0428	MG208172	MG208151	MG207981	MG207999
*Torulafici*		KUMCC 16–0038	MN061341	KY197859	KY197872	KY197879
***Torulagaodangensis***		MFLUCC 17–0234	MF034135	NG 059827	-	-
***Torulagoaensis***		NFCCL 4040	NR 159045	NG 060016	-	-
***Torulaherbarum***		CPC 24414	KR873260	KR873288	-	-
***Torulahollandica***		CBS 220.69	NR 132893	NG 064274	KF443393	KF443401
***Torulahydei***		KUMCC 16–0037	MN061346	MH253926	-	MH253930
***Torulamackenziei***		MFLUCC 13–0839	MN061344	KY197861	KY197874	KY197881
***Torulamasonii***		CBS 245.57	NR 145193	NG 058185	-	-
*Torulamasonii*		DLUCC 0588	MG208173	MG208152	MG207982	MG208000
*Torulamasonii*		KUMCC 16–0033	MN061339	KY197857	KY197870	KY197877
***Torulapluriseptata***		MFLUCC 14–0437	MN061338	KY197855	KY197869	KY197875
***Torulapolyseptata***		KUMCC 18–0131	MK071671	MK071673	-	MK077674
*Torula* sp.		CBS 246.57	KF443411	KR873290	-	-
*Torulalancangjiangensis*		HKAS 112709	NR 175706	MW879526	MW729780	MZ567104
***Torulathailandica***		GZCC 20–0011	MN907426	MN907428	-	-
***Torulacanangae***		MFLUCC 21–0169	OL966950	OL830816	-	ON032379
***Torulachinensis***		UESTCC 22.0085	OQ127986	OQ128004	-	-
***Torulalongiconidiophora***		UESTCC 22.0088	OQ127983	OQ128001	OQ158967	OQ158972
*Torulalongiconidiophora*		UESTCC 22.0125	OQ127984	OQ128002	OQ158972	OQ158972
***Torulaphytolaccae***		ZHKUCC 22-0107	ON611796	ON611800	ON660879	ON660881
*Torulaphytolaccae*		ZHKUCC 22-0108	ON611795	ON611799	ON660878	ON660880
***Torulasichuanensis***		UESTCC 22.0087	OQ127981	OQ127999	-	-
***Torulasubmersa***		UESTCC 22.0086	OQ127985	OQ128003	OQ158968	OQ158972
*Torulasubmersa*		KUNCC 22–12426	OQ991910	OQ991917	-	OQ999003
*Cylindrotorulaindica*		NFCCI 4836	NR 175156	NG 081308	MT321490	MT321492
*Cylindrotorulaindica*		NFCCI 4837	MT339445	MT339443	MT321491	MT321493
***Dendryphionaquaticum***		MFLUCC 15–0257	KU500566	KU500573	-	-
***Dendryphioncomosum***		CBS 208.69	MH859293	MH871026	-	-
*Dendryphioneuropaeum*		CPC 22943	KJ869146	KJ869203	-	-
***Dendryphioneuropaeum***		CPC 23231	KJ869145	KJ869202	-	-
*Dendryphionfluminicola*		KUMCC 15–0321	MG208160	MG208139	MG207971	MG207990
*Dendryphionfluminicola*		DLUCC 0849	MG208161	MG208140	MG207972	MG207991
***Dendryphionfluminicola***		MFLUCC17–1689	NR 157490	MG208141	-	MG207992
***Dendryphionhydei***		KUMCC 18–0009	MN061343	MH253927	-	MH253931
*Dendryphionnanum*		HKAS84010	KU500568	KU500575	-	-
*Dendryphionnanum*		HKAS84012	KU500567	KU500574	-	-
*Dendryphionnanum*		MFLUCC 16–0987	MG208156	MG208135	MG207967	MG207986
***Dendryphionsubmersum***		MFLUCC15–0271	KU500565	KU500572	-	-
*Dendryphionsubmersum*		KUMCC15–0455	MG208159	MG208138	MG207970	MG207989
***Neotorulaaquatica***		MFLUCC 15–0342	KU500569	KU500576	-	-
***Neotorulasubmersa***		HKAS 92660	NR 154247	KX789217	-	-
*Neopodoconisaquaticum*		KUMCC 15–0297	MG208165	MG208144	MG207975	MG207995
***Neopodoconisaquaticum***		MFLUCC 16–1113	MG208164	MG208143	MG207974	MG207994
***Neopodoconispandanicola***		KUMCC 17–0176	MH275084	MH260318	MH412759	MH412781
***Neopodoconiscangshanense***		MFLUCC 20–0146	MW010284	MW010281	MW012636	-
*Neopodoconiscangshanense*		MFLUCC 20–0147	MW010285	-	-	-
***Rutolagraminis***		CPC 33267	MN313814	MN317295	-	-
*Rutolagraminis*		CPC 33695	MN313815	MN317296	-	-
*Rutolagraminis*		CPC 33715	MN313816	MN317297	-	-
***Neopodoconisthailandica***		MFLUCC 13–0840	MN061347	NG 059703	KX437761	KX437766
*Neopodoconisthailandica*		KUMCC 16–0012	MN061348	KX437758	KX437762	KX437767

**Table 2.** Checklist of Torulaceae species from freshwater habitats in China.

Species	Distribution		Habitat	New species/record		Reference
*Dendryphionaquaticum*		Yunnan	Lotic		new species	Su et al. (2016)
*Dendryphionfluminicola*		Yunnan	Lotic		new species	Su et al. 2018
*Dendryphionnanum*		Yunnan	Lotic		new record	Su et al. (2016)
*Dendryphionsubmersum*		Yunnan	Lotic		new species	Su et al. (2016)
*Neotorulaaquatica*		Yunnan	Lotic		new species	Su et al. (2016)
*Neotorulasubmersa*		Yunnan	Lotic		new species	Hyde et al. (2016)
*Neopodoconisaquaticum*		Yunnan	Lotic		new species	Su et al. (2018)
*Neopodoconiscangshanensis*		Yunnan	Lotic		new species	Qiu et al. (2022)
*Neopodoconispandanicola*		Yunnan	Lotic		new record	Qiu et al. (2022)
*Torulaaquatica*		Yunnan	Lentic and Lotic		new species	Su et al. (2018)
*Torulafici*		Yunnan	Lotic		new record	Su et al. (2018)
*Torulalancangjiangensis*		Yunnan	Lotic		new species	Boonmee et al. (2021)
*Torulamackenziei*		Yunnan	Lotic		new record	Boonmee et al. (2021)
*Torulagaodangensis*		Guizhou	Lotic		new species	Hyde et al. (2020)
*Torulachinensis*		Sichuan	Lotic		new species	Tian et al. 2023
*Torulalongiconidiophora*		Sichuan	Lotic		new species	Tian et al. 2023
*Torulasichuanensis*		Sichuan	Lotic		new species	Tian et al. (2023)
*Torulasubmerse*		Sichuan	Lotic		new species	Tian et al. 2023
*Torulamasonii*		Yunnan	Lentic		new record	Su et al. (2018)
